# Supplementary material for: A sensitive high repetition rate arrival time monitor for X-ray free electron lasers
Source: Nat Commun. 2023 Apr 29;14:2495. doi: 10.1038/s41467-023-38143-y (PMC10148857; doi:10.1038/s41467-023-38143-y)
Supplement: Supplementary file 1 — Supplementary Information [file 41467_2023_38143_MOESM1_ESM.pdf]

# Supplementary Information to

# A sensitive high repetition rate arrival time monitor for X-ray Free Electron Lasers

**Michael Diez**[1,2*], **Henning Kirchberg**[3,4], **Andreas Galler**[1*], **Sebastian Schulz**[5], **Mykola Biednov**[1], **Christina Bömer**[1,5], **Tae-Kyu Choi**[1,6], **Angel Rodriguez-Fernandez**[1], **Wojciech Gawelda**[1,7,8], **Dmitry Khakhulin**[1], **Katharina Kubicek**[1,2,9], **Frederico Lima**[1], **Florian Otte**[1], **Peter Zalden**[1], **Ryan Coffee**[10,11], **Michael Thorwart**[2,3], and **Christian Bressler**[1,2,9,*]

[1]European XFEL GmbH, Holzkoppel 4, 22869 Schenefeld, Germany
[2]The Hamburg Centre for Ultrafast Imaging, Luruper Chaussee 149, 22761 Hamburg, Germany
[3]I. Institut für Theoretische Physik, Universität Hamburg, Notkestr. 9, 22607 Hamburg, Germany
[4]Department of Chemistry, University of Pennsylvania, Philadelphia, Pennsylvania 19104, USA
[5]Deutsches Elektronen-Synchrotron DESY, Notkestraße 85, 22607 Hamburg, Germany
[6]XFEL division, Pohang Accelerator Laboratory, Jigok-ro 127-80, 37673 Pohang, Republic of Korea
[7]Faculty of Physics, Adam Mickiewicz University, ul. Uniwersytetu Poznańskiego 2, 61-614 Poznań, Poland
[8]Department of Chemistry, Faculty of Sciences, Universidad Autónoma de Madrid, 28049 Madrid, and IMDEA-Nanociencia, Calle Faraday 9, 28049 Madrid, Spain
[9]Fachbereich Physik, Universität Hamburg, Notkestraße 9-11, 22607 Hamburg, Germany
[10]SLAC National Accelerator Laboratory, 2575 Sand Hill Rd., Menlo Park, CA 94025, United States of America
[11]The Pulse Institute, SLAC National Accelerator Laboratory, Menlo Park, CA 94028, United States of America
[*]corresponding authors: michael.diez@xfel.eu, andreas.galler@xfel.eu, christian.bressler@xfel.eu

## Supplementary Note 1: Heat Load on the Sample

We underline the severity of the heat load deposited in the sample at MHz repetition rate XFEL facilities with a finite element simulation. For the finite element simulation of the heat transfer in materials, the two-dimensional diffusion equation is solved by using the Forward Euler Method[1,2]. The two-dimensional diffusion equation is

$$\frac{\partial U}{\partial t} = D\left(\frac{\partial^2 U}{\partial x^2} + \frac{\partial^2 U}{\partial y^2}\right), \tag{S1}$$

where $D$ is the thermal diffusivity $D = \frac{\lambda}{\rho c_p}$ with the temperature dependent material constants thermal conductivity $\lambda$, density $\rho$ and specific heat capacity $c_p$. By using the finite difference approximation the state of the system at time step $n+1$ may be calculated as[2]

$$U_{ij}^{(n+1)} = U_{ij}^{(n)} + D\Delta t\left[\frac{U_{i+1,j}^{(n)} - 2U_{i,j}^{(n)} + U_{i-1,j}^{(n)}}{(\Delta x)^2} + \frac{U_{i,j+1}^{(n)} - 2U_{ij}^{(n)} + U_{i,j-1}^{(n)}}{(\Delta y)^2}\right]. \tag{S2}$$

The temperature dependant specific heat capacities are assumed to follow the Debye model and the following Debye temperatures where used for the materials: $\Theta D_{diamond}$=1860 K[3], $\Theta D_{Si}$=640 K[4], $\Theta D_{Si_3N_4}$=1145 K[5]. The temperature dependent thermal conductivity can be found in literature for CVD diamond[3], silicon[6] and $Si_3N_4$[7]. From these references the thermal conductivity was digitised to be implemented in our simulation.
The heat diffusion in silicon (Si), silicon nitride ($Si_3N_4$), yttrium aluminium garnet (YAG) and Diamond (C) is calculated after a 4.5 MHz X-ray pulse train with up to 2700 pulses hitting the sample. The simulation considers the temperature dependence of the specific heat capacity $c_p$ and temperature dependant thermal conductivity $\lambda$ of the materials. The usual X-ray pulse duration of well below 100 femtoseconds is short compared to phonon life times, such that in a first approximation no thermal conduction will take place during the pulse duration[8]. Photoelectrons generated by the X-ray photons will carry their energy only over distances of less than a micrometer which is short compared to the attenuation lengths of X-ray photons in materials[9] and mostly smaller compared to the FWHM diameter of mostly used X-ray focal sizes. Under the assumption that the X-ray pulse energy

along the X-ray beam axis through the sample is equally absorbed, the initial heat delivered by an X-ray pulse in the sample along the X-ray axis is uniform. In this case, the heat diffusion can be reduced to a two-dimensional problem, where only heat diffusion perpendicular to the X-ray beam axis occurs in the material. The finite element simulation is based on thermal diffusion in a 2d-model[1] and solves the heat diffusion equation for these materials with a finite difference approximation[2], described in the Methods section of the main text.

The results of the simulation clearly indicate why diamond is a superior candidate for MHz repetition rate beam diagnostics compared to silicon and silicon nitride. In Fig. S1 a) the temperature evolution of the hottest volume fraction (center of a Gaussian beam profile) in the four different materials is simulated after the absorption of one 10 keV X-ray pulse with a FWHM beam diameter of 100 μm and a pulse energy of 20 μJ in the respective sample with a thickness of 50 μm. Due to the rather high X-ray photon absorption cross-section, YAG absorbs with 51% the highest fraction of the pulse energy, followed by silicon and silicon nitride with 31% and 30% respectively. Because of the X-ray absorption cross-section and the lowest heat capacity, YAG is heated up the most by a single X-ray pulse and increases its temperature by $\Delta T=7.3$ K. Despite almost identical X-ray absorption cross-sections and heat capacities at room temperature, the X-ray-induced temperature in silicon and silicon nitride is different due to different densities of the materials. The increase in temperature after the partial absorption of an X-ray pulse, with the aforementioned properties, is $\Delta T=6.6$ K for silicon and $\Delta T=4.7$ K for silicon nitride. The temperature increase in diamond is, according to this simulation, only $\Delta T=0.6$ K. The very small temperature increase in diamond is due to the much smaller X-ray absorption cross-section, absorbing only 2% of the X-ray pulse energy. After the initial temperature increase, the heat diffusion and subsequent cooling process in the materials is simulated with the finite difference method over a time span of 100μs.

When heated up, the heat transport and therefore the heat dissipation in diamond is tremendously faster than in other materials and the cooling process is therefore faster. For a realistic simulation, we assumed that the sample is placed behind a diamond grating in the first diffraction order where roughly 1 percent of the original pulse energy is delivered to the sample, which is approximately 20 μJ. For the simulation, the spacing between the single X-ray pulses is 222 ns, equaling the full EuXFEL 4.5 MHz repetition rate. The temporal evolution of the sample volume in the center of a Gaussian shaped X-ray beam over the time span of 200 μs (900 pulses) is simulated in Fig S1 b). The combination of lower specific heat capacities and shorter attenuation lengths of YAG, Si and $Si_3N_4$, yields a multiple times higher temperature increase with each single X-ray pulse than in diamond. Together with the worse thermal conductivity of these materials, this leads to a drastic increase of the overall temperature in these materials, which ultimately leads to the reaching of their individual melting point already. When reaching their individual melting point, the simulations were stopped. Under these conditions, silicon reaches its melting point of 1687 K after 464 X-ray pulses. Silicon nitride reaches its melting point of 2173 K after 985 X-ray pulses and YAG already reaches its melting point of 2213 K already after 372 pulses. Despite the superior combination of longer X-ray attenuation length and higher thermal conductivity, the temperature of diamond increases only slowly, reaching an equilibrium at around 305 K for these X-ray pulse conditions. The inset of Fig S1 c) shows the temperature increase in the materials for the first 10 pulses.

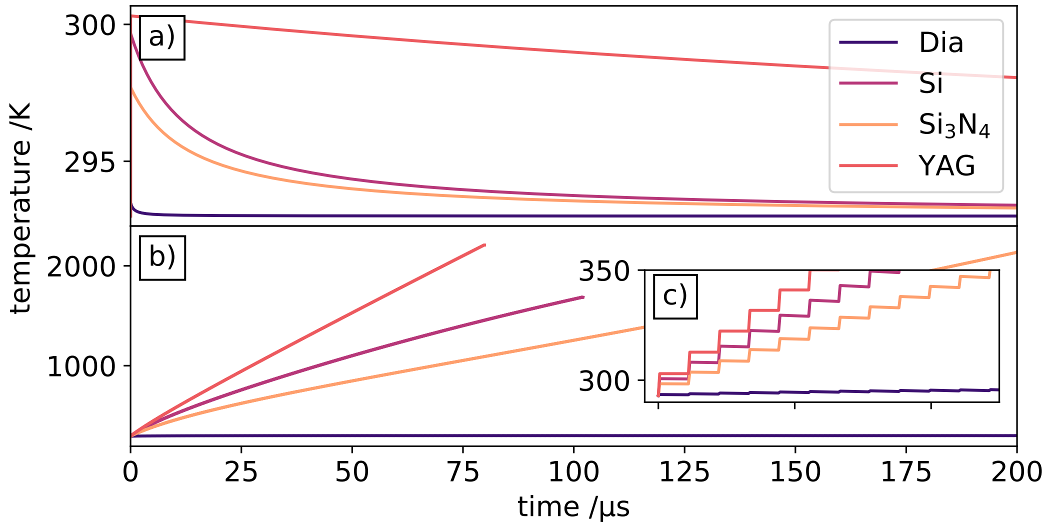


**Figure S1.** Simulated heat propagation in Diamond, Si and $Si_3N_4$. The heat dissipation from 600 K to room temperature is simulated in a). In b) the simulation shows the simulated heat evolution in the different samples for the X-ray beam properties used in the experiment, for a full 2700 pulses EuXFEL pulse train.

## Supplementary Note 2: Self-Referenced Signal Simulation

The starting point of the simulation is a bandwidth-limited Gaussian laser pulse with a central frequency of 749 THz (400 nm) and a FWHM bandwidth of 45 THz (25 nm). The pulse in the spectral domain is fully described via

$$E(\omega) = A\, \mathrm{e}^{-\frac{(\omega-\omega_0)^2}{4\sigma\sqrt{2\,\ln\,2}}}, \tag{S3}$$

with the amplitude $A$, angular frequency $\omega = 2\pi$f, the central angular frequency of the laser pulse $\omega_0$ and the FWHM bandwidth $\sigma$. The spectrum of the pulse is defined as $|E(\omega)|^2$. The spectral domain and the electric field in the time domain are related by the Fourier inverse theorem $\mathscr{F}^{-1}(E(\omega)) = E(t)$ and $\mathscr{F}(E(t)) = E(\omega)$. The bandwidth-limited pulse is shown in Fig. S2 a) in the time domain and a') in the spectral domain. This pulse is linearly propagated through a 100 mm thick block of SF11 glass to chirp the pulse. The linear propagation of a laser pulse influences the spectral phase $\phi(\omega)$ of the pulse and may be expressed as[10]

$$E(\omega, z) = E(\omega, z=0)\, e^{-ik(\omega)z}, \tag{S4}$$

where $E(\omega, z=0)$ describes the original pulse entering the dispersive media at position z=0 and $k(\omega) = \pm\omega n(\omega)/c_0$ the dispersion relation. To account for dispersive effects of the optical media, the dispersion relation is usually expressed as a Taylor expansion

$$k(\omega) = k_0 + \frac{\partial k}{\partial \omega}(\omega-\omega_0) + \frac{1}{2}\frac{\partial^2 k}{\partial \omega^2}(\omega-\omega_0)^2 + \frac{1}{6}\frac{\partial^3 k}{\partial \omega^3}(\omega-\omega_0)^3 + \ldots \tag{S5}$$

In the simulation, effects up to the third order are taken into account. These three terms are adding additional phase to the original pulse. The first term, linear in frequency, adds a constant time delay (GV), the second term symmetrically broadens the pulse in time (GVD) and the third term asymmetrically broadens the pulse in time (TOD). The full simulation with the propagation of the initial laser pulse through the birefringent crystals (BC) creating the time-sheared polarisation components (PC), through the diamond sample applying the X-ray-induced refractive index change and through the second polariser is shown in Fig. S2. The application of the X-ray-induced refractive index change and the correlating phase shift and amplitude change of the two transmitted PCs in the interaction sample are exemplarily illustrated in Fig. S3. The change of the complex refractive index is applied by transforming the optical pulse from the spectral $E(\omega)$ into the temporal domain $E(t)$ using the Fourier Transformation. The real part of the X-ray induced refractive index change induces a time dependant phase shift $\phi(t)$ in the transmitted chirped optical pulse, while the imaginary part alternates the amplitude of the transmitted optical pulse. (Note: The X-ray induced change of the imaginary part is negligible in diamond, hence no X-ray induced absorption can be observed) The resulting X-ray arrival-time spectrum is heavily influenced by the amount of the X-ray-induced phase shift. The steepness

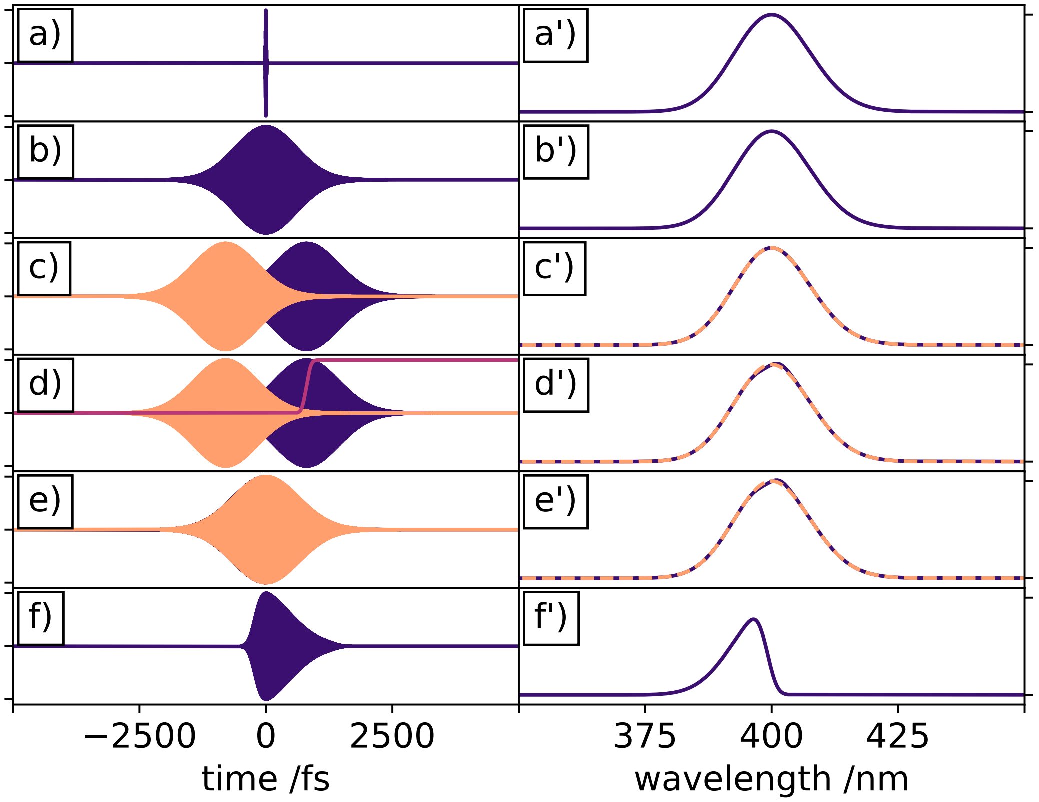


**Figure S2.** Self-referenced timing-tool simulation: Electric field (a) and power spectrum (a') of the initial laser pulse. This laser pulse propagates through the additional glass block to stretch it in time, accumulating chirp (b) with an unchanged power spectrum (b'). The first BC creates two time-sheared PCs (c) with identical spectra (c'). The X-ray-induced refractive index change adds a phase shift to both PC (d) and is applied as a Gaussian error-function (purple). For small phase shifts both PC spectra are nearly unchanged, but with higher phase shifts self-phase modulation effects can occur (d'). The second BC combines both PCs temporally (e) without changing their spectra (e'). Due to the X-ray-induced phase shift parts of the laser pulse can be transmitted through the second polariser (f), delivering the self-referenced arrival-time spectrum (f') with a cut-off edge indicating the X-ray arrival-time.

of the cut-off edge is of course influenced by the time duration over which the phase-shift occurs. The main influence of the phase shift is on the amplitude of the resulting arrival-time spectrum. The amplitude correlates with the phase-shift value (Fig. S4). Small X-ray-induced phase shifts (between 0° and roughly 20°) only affect the amplitude of the arrival-time spectra (Fig. S4 b). At larger phase shift angles, up to 360°, additional effects are changing the shape of the arrival-time spectra (Fig. S4 c). Cross-phase modulation[11] (CPM)-like effects start to show their effect in this phase shift range and cause different spectral changes to the two time-sheared PCs. Therefore, the resulting arrival-time spectra shapes change with increasing phase shifts (c) and the arrival-time edge position changes its spectral position. For phase shifts larger than 360°, the spectra become very complex. For each further full cycle shifted (n × 360°), a minimum in the arrival-time spectrum appears (d). This is caused by the fact that the refractive index is not changed instantly but over a time span of up to hundreds of fs, depending on the X-ray photon energy. In this process, each time when the X-ray-induced phase shift is exactly 360°, the involved spectral regions of both PCs are perfectly synchronized behind the second BC and will recreate the original 45○ polarization, and thus will not be transmitted through the second polariser. If the refractive index change is large enough to shift the phase by *n* cycles, an equal amount of minima will be observed in the spectrum.

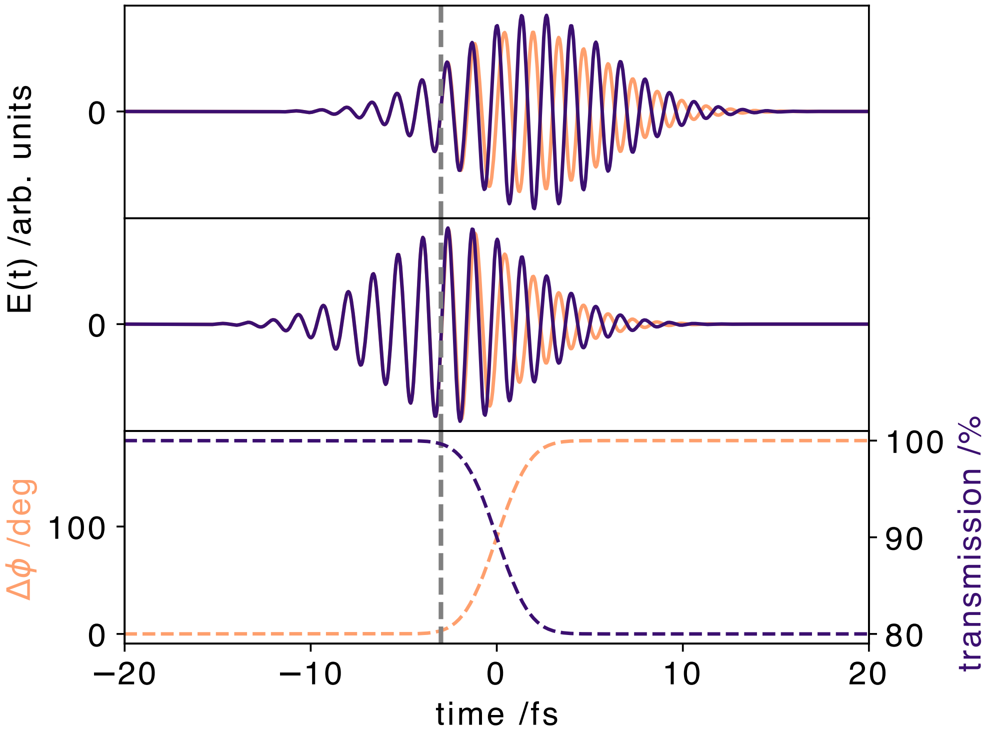


**Figure S3.** Application of the X-ray-induced refractive index change to the two PCs. The grey vertical line indicates the X-ray arrival-time where the phase shift and amplitude change is applied to the trailing (top) and leading (middle) PC. These changes are included by a Gaussian-like error-function shown on the bottom. In this case the phase shift is set to 180° and the amplitude change to 20% over a time of roughly 10 fs.

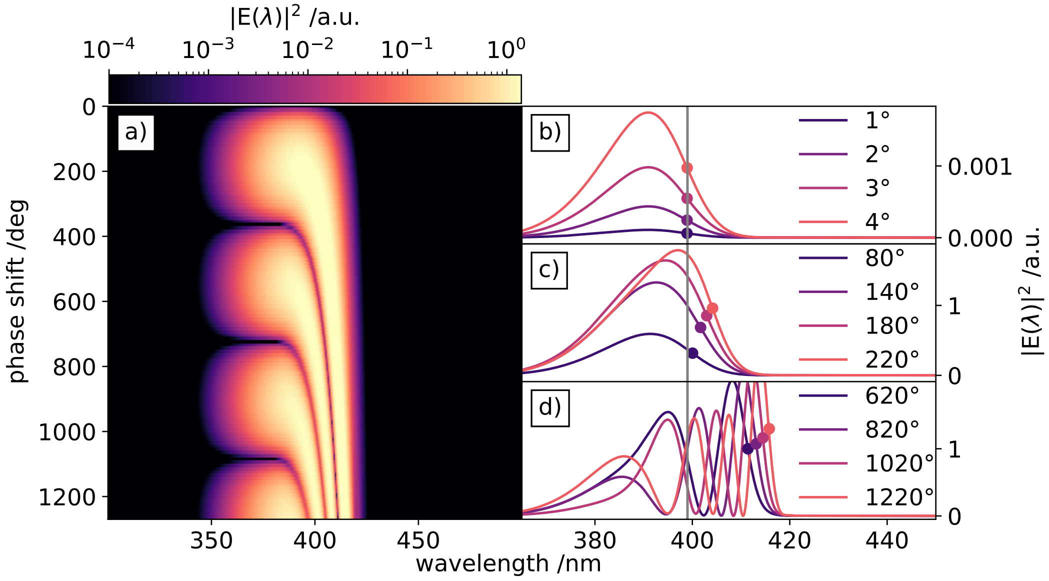


**Figure S4.** Dependence of resulting X-ray arrival-time spectra on the X-ray-induced phase shift. A 2d-intensity image is illustrated in a), showing arrival-time spectra for identical X-ray arrival-times, but different X-ray-induced phase shifts from 0° to 1280°. The amplitudes of the spectra are color-coded using a logarithmic color map. Individual spectra for specific phase shift values are shown in b), c) and d). The gray vertical line indicates the cut-off edge position for arrival-time spectra with small X-ray-induced phase shifts. For large X-ray-induced phase shifts, the cut-off edge is slowly shifting (c) and the overall spectrum becomes quite complex (d).

## Supplementary Note 3: Time Axis Calibration

Fig. S5 displays two exemplary arrival time spectra, which were fitted by a broadened step function from the zero line to the left and up to the maximum of the spectral signals. The fit function used to determine the edge position of the self-referenced signal is a Gaussian error function as a function of detector pixel $x$:

$$f(x) = F\left(1+\mathrm{erf}\left(\frac{\mathrm{x}-\mu}{\sigma}\right)\right). \tag{S6}$$

The fitting parameters for the Gaussian error function are its centre of the step $\mu$ and width of the step $\sigma$. The amplitude $F$ of the fitting function is fixed to the maximum of the self-referenced arrival-time spectrum. It thus describes the self-referenced signal from its onset to its cut-off. The position $\mu$ of the half-rise value of the error function fit serves to define the precise arrival time of the X-ray pulse. The conversion of pixel position (or wavelength) to arrival time is then achieved with a pixel-to-time calibration: With a motorized optical delay stage, we changed the relative time delay between optical and X-ray pulses in steps of 200 fs and measured hundreds arrival-time spectra for each time delay (Fig. S6 a). The pixel position of each arrival-time spectrum was analysed for all optical delay stage positions and their central time delay position extracted (via their mean average value) for each set time delay. A linear fit function ($y = mx + b$) is then used to determine the pixel-to-time calibration for this measurement (Fig. S6 b), where the parameters $m$ and $b$ describe the slope and offset of the linear term. The very narrow bandwidth of only 20 nm FWHM for the optical pulse indeed justifies the linear approximation of the chirp, while for broader bandwidth optical pulses, a quadratic approximation would be applied. For this calibration procedure we used the more accurate optical synchronisation scheme of EuXFEL to retrieve the pixel-to-time calibration relationship, yielding the (28±5) fs/pixel.

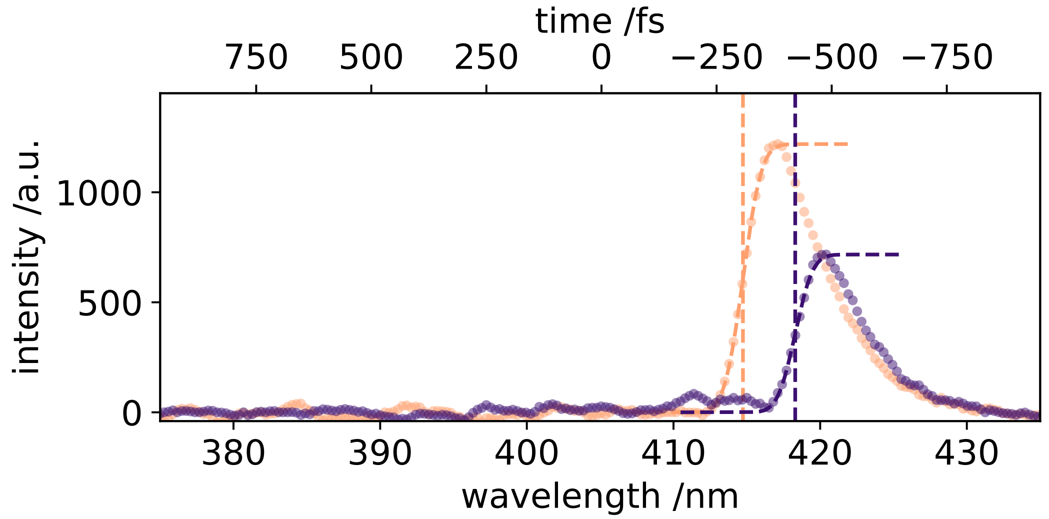


**Figure S5.** Two arrival-spectra at different temporal delays. The step-like error-functions are fitted to the raw data (solid points) and are shown as solid lines. The determined edge positions are indicated by the vertical dashed lines. The center of the time axis on top was arbitrary set to 0 fs.

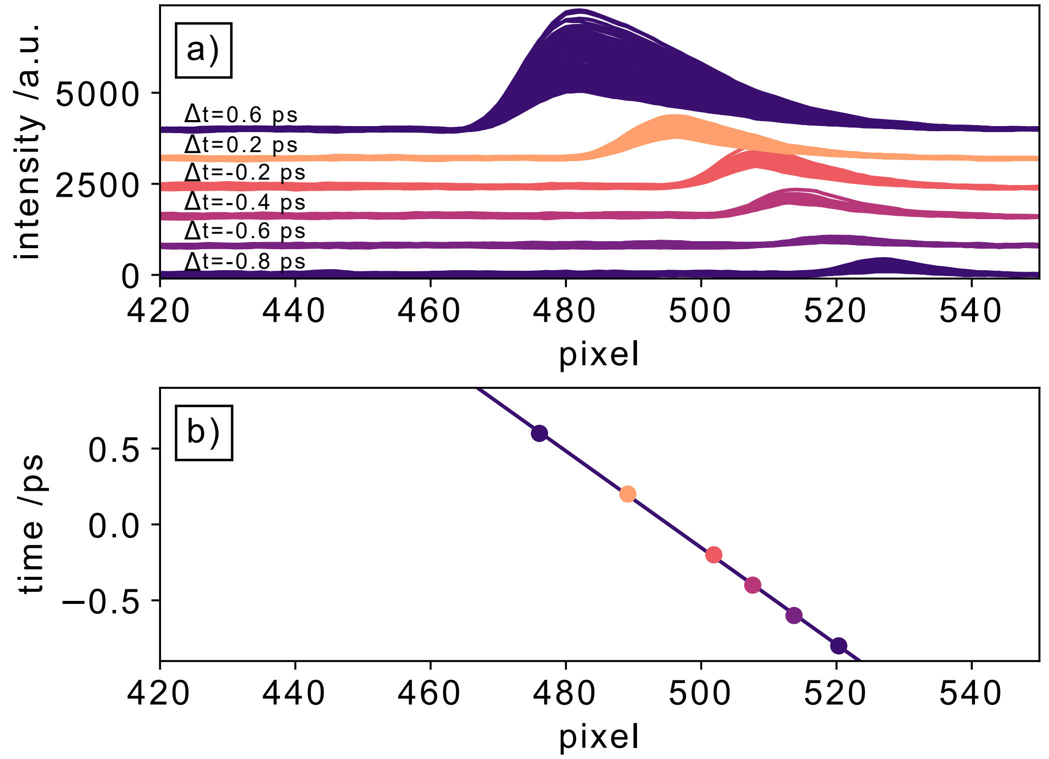


**Figure S6.** Calibration of the time axis. Individual arrival time spectra for different delay stage settings (a). Each color shows thousands of individual arrival-time spectra at a fixed delay between the optical and X-ray pulses. Pixel-to-time calibration with a linear function (b). Each point is the average pixel position of all arrival-time spectra at each delay stage position. The standard deviation for each point is between 0.5 and 1.3 pixel yielding an overall timing accuracy of 19 fs.

## Supplementary Note 4: Spectral Encoding Results

During the measurement campaign we have also included the spectral encoding scheme, which is the accepted method at hard X-ray FELs to determine relative arrival times between laser and XFEL pulses. The spectral encoding data are shown in the figures below (Fig. S7 spectral encoding scheme with an invasive YAG crystal, Fig. S8 spectral encoding scheme with diamond. The horizontal axis in pixels corresponds to wavelength of the chirped laser pulse). While we observed a timing tool signal with the YAG material we could not detect any absorption signal with the spectral encoding scheme in diamond, and can only conclude that its phase sensitivity is not measurable in diamond samples. The physical reason for this behavior is that x-rays produce orders of magnitude less conduction band electrons in diamond than in e.g. YAG. This also illustrates the superiority of our phase-sensitive timing tool.

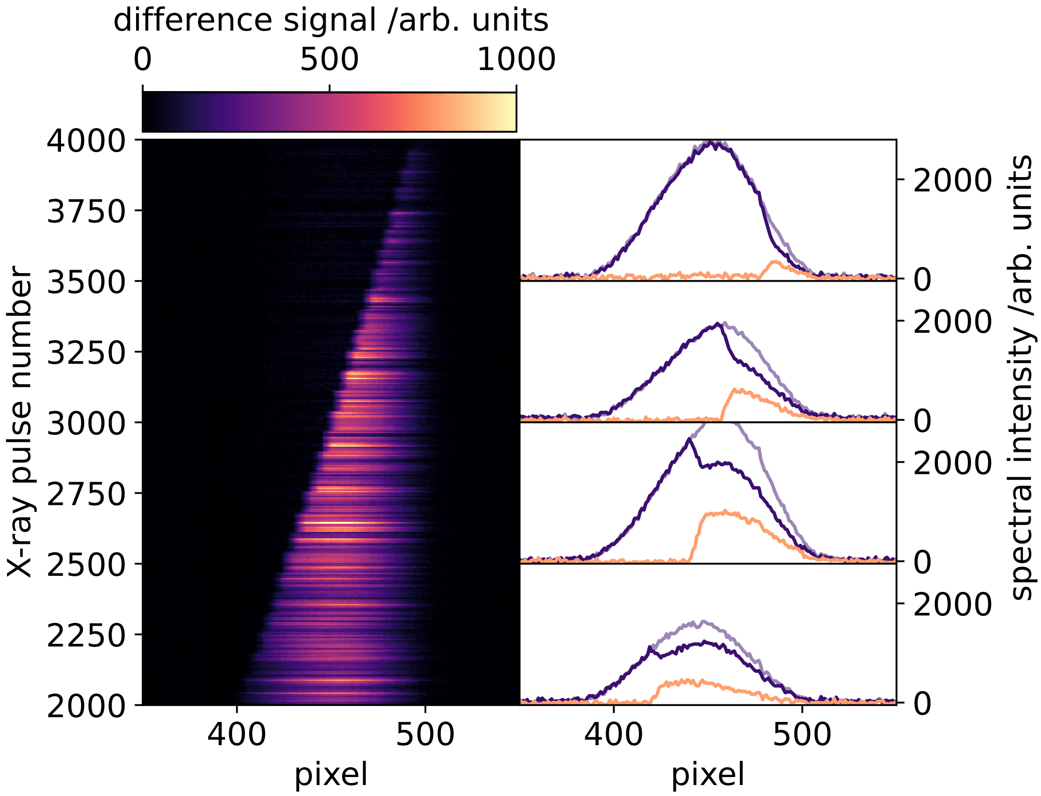


**Figure S7.** Spectral encoding in YAG. The difference signals of a spectral encoding test measurement are shown in the 2-dimensional plot. The relative X-ray arrival time is systematically scanned from early (top) to later (bottom) arrival-times. Four example measurements are extracted on the right-hand side. The actual spectral encoding spectrum is shown in blue, the reference spectrum shown in light blue, and the difference signal with the imprinted X-ray arrival-time is shown in orange.

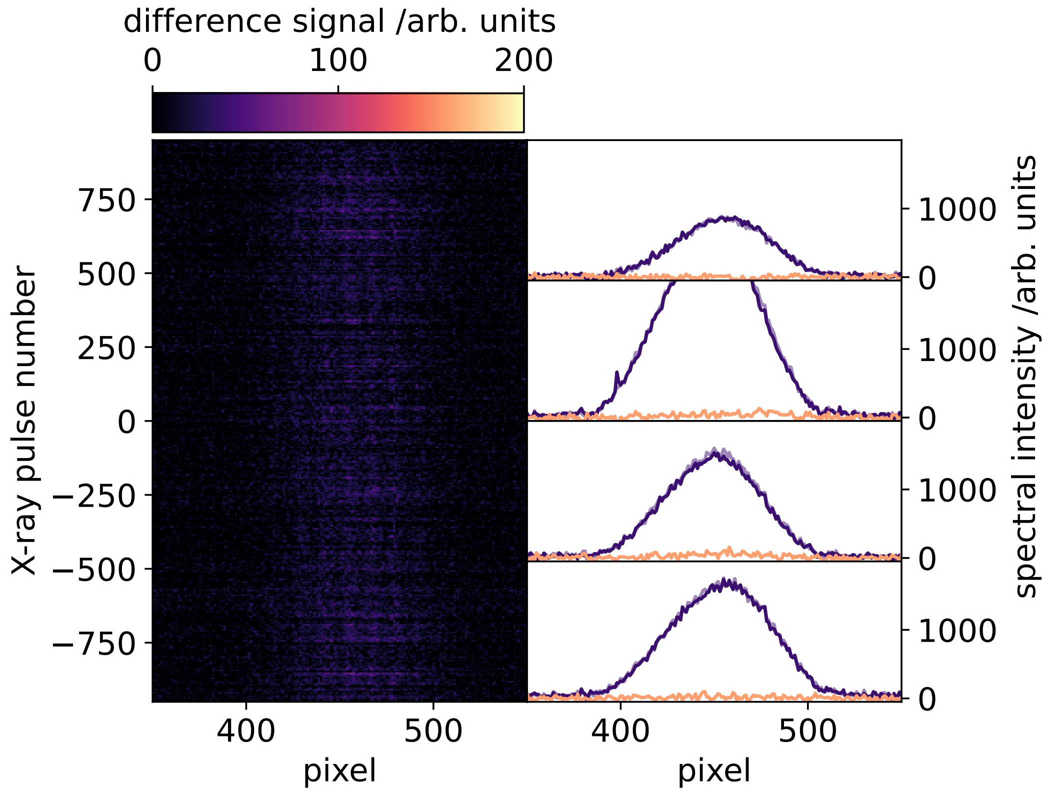


**Figure S8.** Difference spectra of a spectral encoding time-delay scan in diamond. Difference signals of the entire measurements are shown in the left 2-dimensional image. On the four sub-panels on the right, four exemplary measurements are shown with the spectral encoding spectrum (blue), reference spectrum (light blue) and difference signal in orange. No X-ray-induced spectral encoding signal can be observed.

## Supplementary Note 5: Physicals Values used in our Simulations

Here we provide the physical values which we used in our Simulations. The diamond properties used for the Drude model are:

| Diamond properties used for Drude model | |
|---|---|
| $n_{0,400\text{nm}}$ | 2.46[12] |
| $m^*_{\text{el}}$ | 1.56[13] |
| $m^*_{\text{et}}$ | 0.28[13] |
| $m^*_{\text{e}}$ | $(m^2_{\text{et}} m_{\text{el}})^{1/3}$[14] |
| $\omega_{400\text{nm}}$ | 4.709e15 rad/s [calculated] |
| $\mu_{\text{e}}$ | 4500 $\text{cm}^2/\text{Vs}$[14] |
| $\tau_{\text{e}}$ | 1.27 ps [calculated via $\mu_{\text{e}} m^*_e/\text{e}$] |

**Table S1.** Diamond properties used to calculate the refractive index change with the Drude model.

The diamond properties used for the Maxwell-Garnett model are:

| Diamond properties used for Maxwell-Garnett model | |
|---|---|
| $\mu_{9200\text{eV}}$ | 1600 µm[9] |
| $E_{\text{e-h}}$ | 12.2 eV[15] |
| $\varepsilon_{S,d}$ | 7.85[16] |
| $\varepsilon_{S,p}$ | $0.99\varepsilon^{*}_{S,d}$ |
| $\varepsilon_{\infty,d}$ | 5.56[16] |
| $\varepsilon_{\infty,p}$ | $0.99\varepsilon^{*}_{\infty,d}$ |
| $\tau_{D,d}$ | 2.48 µs[16] |
| $\tau_{D,p}$ | $1.5\tau^{**}_{D,d}$ |
| $a$ | $3.567\text{x}10^{-10}$ m[17] |
| $r_p$ | $0.80a^{***}$ |

**Table S2.** Properties used in the simulation
* We assume a small reduction of the overall dielectric constant of 1% within the electronic polaron since only the fast electrons within the dielectric background will respond on the sub-picosecond time-regime. ** Due to the slightly reduced polarisation of the distorted charge clouds within the electronic polaron we estimate an enhanced Debye relaxation time about 1.5 in comparison to the bulk diamond. *** An electronic polaron has an extension around or smaller than the lattice constant, such that we choose its radius about 80% of the lattice constant of diamond.

| Simulation Properties | | | |
|---|---|---|---|
| Constants | Diamond | BBO o | BBO e |
| $n_{400\text{nm}}$ | 2.46 | 1.69 | 1.57 |
| $\text{GVD}_{400\text{nm}}$ ($\text{fs}^2$/mm) | 7 | 209.44 | 150.50 |
| $\text{TOD}_{400\text{nm}}$ ($\text{fs}^3$/mm) | 7 | 778 | 7507 |
| Diameter (mm) | 0.05 | 5 | 5 |

**Table S3.** Properties used in the simulation

## Supplementary References


1. Crank, J. *The mathematics of diffusion* (Clarendon Press, Oxford, [Eng], 1975), 2. edn.
2. Langtangen, H. P. & Linge, S. *Finite Difference Computing with PDEs: A Modern Software Approach*, vol. 16 of *Texts in Computational Science and Engineering* (Springer International Publishing, Cham, 2017).
3. The CVD diamond booklet. https://www.diamond-materials.com/site/assets/files/1095/cvd_diamond_booklet.pdf. Accessed: 2020-06-30.
4. Cerofolini, G. & Meda, L. *Physical Chemistry of, in and on Silicon*, vol. 8 of *Springer Series in Materials Science* (Springer Berlin Heidelberg, Berlin, Heidelberg, 1989).
5. Brown, R. C. & Swaminathan, P. K. Silicon nitride equation of state. *AIP Conf. Proc.* **1793**, 050013, DOI: 10.1063/1.4971547 (2017). https://aip.scitation.org/doi/pdf/10.1063/1.4971547.
6. Glassbrenner, C. J. & Slack, G. A. Thermal Conductivity of Silicon and Germanium from 3K to the Melting Point. *Phys. Rev.* **134**, A1058–A1069, DOI: 10.1103/PhysRev.134.A1058 (1964).
7. Xiang, H., Feng, Z., Li, Z. & Zhou, Y. Theoretical predicted high-thermal-conductivity cubic Si3N4 and Ge3N4: promising substrate materials for high-power electronic devices. *Sci. Reports* **8**, 14374, DOI: 10.1038/s41598-018-32739-x (2018).
8. Torres Alvarez, P. *Phonon Spectrum and Transient Regimes in the KCM*, 117–135 (Springer International Publishing, Cham, 2018).
9. Henke, B., Gullikson, E. & Davis, J. X-Ray Interactions: Photoabsorption, Scattering, Transmission, and Reflection at E = 50-30,000 eV, Z = 1-92. *At. Data Nucl. Data Tables* **54**, 181 – 342, DOI: https://doi.org/10.1006/adnd.1993.1013 (1993).
10. Saleh, B. E. A. & Teich, M. C. *Fundamentals of photonics; 2nd ed.* Wiley series in pure and applied optics (Wiley, New York, NY, 2007).
11. Alfano, R. R., Li, Q. X., Jimbo, T., Manassah, J. T. & Ho, P. P. Induced spectral broadening of a weak picosecond pulse in glass produced by an intense picosecond pulse. *Opt. Lett.* **11**, 626–628, DOI: 10.1364/OL.11.000626 (1986).

**12.** Phillip, H. R. & Taft, E. A. Kramers-Kronig Analysis of Reflectance Data for Diamond. *Phys. Rev.* **136**, A1445–A1448, DOI: 10.1103/PhysRev.136.A1445 (1964).

**13.** Naka, N., Fukai, K., Handa, Y. & Akimoto, I. Direct measurement via cyclotron resonance of the carrier effective masses in pristine diamond. *Phys. Rev. B* **88**, 035205, DOI: 10.1103/PhysRevB.88.035205 (2013).

**14.** Isberg, J. *et al.* High Carrier Mobility in Single-Crystal Plasma-Deposited Diamond. *Science* **297**, 1670–1672, DOI: 10.1126/science.1074374 (2002). https://science.sciencemag.org/content/297/5587/1670.full.pdf.

**15.** Gabrysch, M. *Charge Transport in Single-crystalline CVD Diamond*. Ph.D. thesis, Uppsala University, Electricity (2010).

**16.** Brozdnichenko, A. N., Dolgintsev, D. M. & Castro, R. A. The dielectric properties of the diamond-like films grown by ion-plasma method. *J. Physics: Conf. Ser.* **572**, 012025, DOI: 10.1088/1742-6596/572/1/012025 (2014).

**17.** Hom, T., Kiszenik, W. & Post, B. Accurate lattice constants from multiple reflection measurements. II. Lattice constants of germanium silicon, and diamond. *J. Appl. Crystallogr.* **8**, 457–458, DOI: 10.1107/S0021889875010965 (1975).
